# Antioxidative Properties and Acrylamide Content of Functional Wheat-Flour Cookies Enriched with Wild-Grown Fruits

**DOI:** 10.3390/molecules27175531

**Published:** 2022-08-28

**Authors:** Barbara Borczak, Marek Sikora, Joanna Kapusta-Duch, Maria Fołta, Agnieszka Szewczyk, Gabriela Zięć, Ivo Doskočil, Teresa Leszczyńska

**Affiliations:** 1 Department of Human Nutrition and Dietetics, Faculty of Food Technology, University of Agriculture in Krakow, Al. Mickiewicza 21, 30-149 Krakow, Poland; 2 Department of Carbohydrate Technology, Faculty of Food Technology, University of Agriculture in Krakow, Al. Mickiewicza 21, 30-149 Krakow, Poland; 3 Department of Food Chemistry and Nutrition, Faculty of Pharmacy, Jagiellonian University Medical College, 9 Medyczna St, 30-688 Krakow, Poland; 4 Department of Microbiology, Nutrition and Dietetics, Faculty of Agrobiology, Food and Natural Resources, Czech University of Life Sciences Prague, 165 00 Prague, Czech Republic

**Keywords:** wheat-flour cookies, wild-grown fruits, antioxidant properties, polyphenols, acrylamide

## Abstract

This study aimed to evaluate the impact of widely grown fruits (wild roses, elderberries, sea buckthorns, rowans, chokeberries, and hawthorns) as a functional ingredient in wheat-flour cookie formulation on antioxidative properties with a simultaneous reduction of the carcinogen-like compound acrylamide. The organoleptic features of the cookies were assessed by a panel of consumers. The following parameters were measured: chemical composition, total polyphenols, polyphenolic profile, antioxidant activity, and acrylamide content. The overall ratings of the tested cookies with the addition of chokeberries, hawthorns, sea buckthorns, and elderberries were more than satisfactory, while wild rose and rowan cookies were the most widely accepted and best rated by the panelists. The antioxidant activity of the tested cookies was 1.1–15.22 μmol trolox·g^−1^ dm and 2.46–26.12 μmol Fe (II)·g^−1^ dm as measured by the ABTS and FRAP methods, respectively. All the fruit-enriched cookies had significantly higher antioxidative properties (*p* < 0.05) in comparison to the control cookies, but among the fruit-enriched cookies, there were differences in the quality and quantity of particular polyphenols. The acrylamide content was significantly decreased by 59% (hawthorn), 71% (rowan), 87% (wild rose), 89% (sea buckthorn), 91% (elderberry), and 94% (chokeberry) compared with the control cookies (*p* < 0.05). Cookies enriched with wild-grown fruits could constitute a promising novel snack food.

## 1. Introduction

Confectionery products are popular among consumers. Unfortunately, their relatively high energy value and, at the same time, low nutritional value often lead to the limitation of their consumption in the daily human diet. Not without significance is the presence of some potentially toxic compounds, one of them being acrylamide, which might be formed upon heating (>120 °C) in low-moisture conditions, mainly during frying or baking in the presence of reducing sugars together with certain amino acids, such as asparagine [1]. Acrylamide is included in the State of California’s Proposition 65 list as a carcinogen [1,2,3]; it can be found in cigarette smoke and in many types of food, including bread, cereal, coffee, and cookies [1,2]. Laboratory tests showed that acrylamide in the diet may lead to cancer in animals and potentially increase the tumor risk for humans of all ages [3]. It is essential to take into account that elimination of acrylamide from food products is virtually impossible; however, some efforts should be taken to reduce its amount in a given product. Furthermore, current knowledge indicates that for some food product categories, the acrylamide content might be highly dependent on the variations of some ingredients in the raw materials [2,3]. At the same time, the increasingly broader knowledge of consumers and existing trends for healthy, palatable, and natural functional foods force food producers to introduce modernized products enriched with plant and plant byproducts into the market. This might be beneficial from two points of view: increasing the functional properties of food products and decreasing some of the so-called food-borne toxins, such as acrylamide [4]. In the baking industry, the use of fruits, vegetables, and their processed products is an additional source of either water-insoluble or soluble fiber (pectin) or biologically active substances (polyphenols) which have a proven favorable effect on the human body [5]. An interesting alternative worthy of investigation is the possibility of using some of the naturally occurring and wildly grown edibles commonly found in Europe and Asia, which are often described as medicinal plants. The nutrients of wild plants are comparable and sometimes even better than those of cultivated varieties [6,7,8,9,10,11,12,13,14,15,16,17,18,19,20,21]. Wild-grown fruits are the crops of bushes classified into different families: Rosaceae (*Aronia melanocarpa*, *Crataegus* L., *Sorbus aucuparia* L., *Rosa canina* L.), Elaeagnaceae (*Hippophae rhamnoides* L.), and Adoxaceae (*Sambucus nigra* L.) [6,7,8,9,10,11,12,13,14,15,16]. Berries of these plants differ in shape, color, and nutritional properties. These differences depend on many factors, including variety, environment, climatic conditions, and maturity [6,7,8,9,10,11,12,13,14,15,16]. *Aronia melanocarpa* (chokeberry) comes from the eastern part of North America but has been found and gained its popularity all over the world. The bushes may reach up to 2–3 m and fructify in purplish black berries of spherical shape and astringent taste, covered by a waxy coating [6,7]. The most valuable feature of chokeberries is that they contain polyphenolic compounds such as proanthocyanidins, anthocyanins, flavonoids, and phenolic acids [7]. Chokeberries also contain vitamin B, carotenoids, tocopherols, vitamin C, and vitamin K. They are also a good source of both macroelements (K, Ca, P, Mg, and Na) and some essential microelements (Zn, Fe, Se, Cu, Mo, Cr) [6,7]. *Sorbus aucuparia* L. (rowanberry) is a tree, also known as mountain ash tree, which produces bright orange-red, round-shape fruits with a more or less bitter taste [10,17]. Rowanberries are a traditional cooking ingredient. They can be used as a puree, juice, jelly, or jam [10]. They have a favorable nutritional composition, due in part to ascorbic acid, minerals, and phenolic contents. They are also abundant with potassium, calcium, phosphorus, and unsaturated fatty acids [17]. *Crataegus* L. (hawthorn) is a branched shrub/small tree belonging to the Rosaceae family and the Maloideae subfamily [18]. It gives red berries of 10 mm when ripened, containing one seed, which is to be removed before consumption [9]. In many places, the fruits are used in jam preparations and as flavoring for dishes from white meats [18]. Hawthorns are rich in carbohydrates, organic acids, vitamins, minerals and contains more than 150 phenolic compounds including procyanidins, flavonoids, and triterpenoid acid [9,18]. So far, many beneficial properties of hawthorns have been evidenced, for example, as a preventative agent against hypertension, angina, heart failure, cardiac arrhythmias, myocarditis, arteriosclerosis, insomnia, diarrhea, urinary retention, and some intestinal dysfunctions [9,18]. *Rosa canina* L. (wild rose), also called dog rose, is a plant that is a rich source of biologically active compounds (vitamin C, E, A, B1, B2, K, minerals, polyphenolics) having antioxidant, antimutagenic, and anticarcinogenic properties [12,19]. Wild rose berries are oval-shaped elongated or spherical with purplish-red or coral color and a shiny hard skin [12]. They have found broad application in the form of teas, marmalades, jellies, jams, soups, food supplements, nectars, or wine and as an ingredient of probiotic drinks and yoghurts [12,19]. The use of wild rose fruits in the cosmetic industry and as pharmaceuticals has become very popular. It has been evidenced that wild rose berries not only act effectively against common cold, gastrointestinal disorders, infections, various inflammatory diseases, and chronic pains, but also may have anti-obesity, antidiabetic, antinociceptive, antiulcerogenic, and antiproliferative properties [12,19]. The popular names of *Sambucus nigra* L. (elderberry) include elder, elderberry, black elder, European elder, European elderberry, and European black elderberry [16]. It is a vigorously spreading shrub/tree reaching up to 3–10 m [15]. Elderberries are glossy black, with red juicy flesh characterized by a slightly bland but sweet taste [15,16,20]. They are a great source of anthocyanins, organic acids (acetic, malic, valerian, shikimic, tartaric, and benzoic acids), monosaccharides (glucose, fructose), pectins (mainly protopectin), vitamins (A, C, P, and B vitamins), folic acid, and, in smaller amounts, mineral salts. Elderberry is also a source of whole proteins, which include sixteen amino acids, nine of which are essential [16]. The major fatty acids are polyunsaturated fatty acids, present mainly in seeds: α-linolenic, linoleic, and oleic acid [15,16,20]. The evidenced health-promoting properties of elderberry fruits are impressive and include antioxidant, anti-inflammatory, anticancer, anti-influenza, antimicrobial, antidiabetic, cardiovascular protective, neuroprotective, and skin-protective activities [16]. The fruits are also used as a natural dye due to their high anthocyanin content. They could be added to fruit wines, marmalades, and jams. In households, elderberries are used to make juices that can be used as a condiment for meat sauces, as well as jams, preserves, marmalades, and syrups [20]. *Hippophae rhamnoides* L. (sea buckthorn) is a bush growing up to 5 m high, cultivated for both food and medicinal purposes. Sea buckthorn fruits are orange-colored and spherical in shape [13,14]. They taste sour and have a peculiar odor, resulting from the presence of 45 volatile compounds: alcohols, esters, ketones, aldehydes, and terpenes, often described as the aroma of an exotic fruit, citrus, or even pineapple. Sea buckthorn berries contain multiple vitamins (A, B1, B2, B3, B6, C, E, K), as well as organic acids, quercetin, flavonoids, macroelements and microelements, and fatty oil, which consists of triacylglycerols with saturated and unsaturated fatty acids (palmitoleic, oleic, linoleic, linolenic). Previously, it was proved that sea buckthorn berries and their derivatives possess a wide range of beneficial characteristics: anti-inflammatory, antitumor, antioxidant, anti-atherosclerotic, cholesterol-lowering, hypoglycemic, hepatoprotective, and antidiabetic [21]. The abovementioned health-promoting properties of wild-grown fruits have long been used in folk medicine and have now found application in the pharmaceutical industry [9,15]. Their application as functional ingredients in the production of wheat-flour cookies has not been tested recently. So far in the literature, enrichment of cakes and cookies with other fruits and fruit byproducts, such as apple pomace [22,23,24], berry pomace [25,26], sour cherry pomace [13,27], mango peel [28], banana peel [29], green banana flour [30], pumpkin and carrot pomace [31], pomegranate peel [32], citrus peel powder [33], grapefruit and citrus fiber [20,21,34,35], potato peel, [36] and watermelon rind [37], has been proposed. Therefore, the aim of this study was to investigate the antioxidant properties of wheat-flour cookies fortified with the abovementioned wild-grown fruits, as well as their reducing effect against acrylamide, together with the organoleptic assessment which might reflect their future acceptance by potential consumers.

## 2. Results and Discussion

In this study, seven types of wheat cookies were tested: (1) wheat-flour cookies without addition of lyophilized fruits (control), (2) wheat-flour cookies with 5% of sea buckthorns (*Hippophae rhamnoides* L.); (3) cookies with 5% of elderberries (*Sambucus nigra* L.); (4) cookies with 5% of hawthorns (*Crataegus* L.); (5) cookies with 5% of rowans (*Sorbus aucuparia* L.); (6) cookies with 5% of chokeberries (*Aronia melanocarpa*); and (7) cookies with 5% of wild roses (*Rosa canina* L.) (Table 1).

### 2.1. Organoleptic Evaluation

Cookies with the addition of wild roses were rated best, obtaining 4.40 points on a 5-point scale (*p* < 0.05) (Table 2). Rowan cookies also received a high score (4.19 points) and did not differ in a statistically significant way from the control cookies (4.14 points) (*p* > 0.05). The overall rating of the other cookies, with the addition of chokeberries (3.90 points), sea buckthorns (3.79 points), elderberries (3.71 points) and hawthorns (3.45 points), was lower in a statistically significant way (*p* < 0.05) but received more than satisfactory assessment. In the literature, the inclusion of fruit byproducts from different plant sources has led to a reduction in the acceptability of the cookies, which was mainly attributed to the bitter flavors present in citrus peels [33] and has also been related to the changes in their color (detected in the majority of the studies) [5]. In this study, the overall appearance and shape were rated worse in the case of chokeberry, hawthorn, sea buckthorn, and elderberry cookies in comparison to the other ones (*p* < 0.05), while the consistency and the surface were rated lower for hawthorn and elderberry cookies (Table 2). An interesting point is that the color and taste of all the fruit-enriched cookies were similarly approved by the panelists, while the flavor of hawthorn cookies was not accepted as much as for the other tested cookies (*p* < 0.05).

### 2.2. Chemical Composition

The results of the chemical composition studies are presented in Table 3.

To the best of our knowledge, there are no data in the literature concerning the effect of wild-grown fruit additions to wheat-flour cookies. The obtained results are in concordance with the data presented in the literature for wheat-flour cookies in the case of dry matter (93.08–97.13 g·100 g^−1^), carbohydrates (49.22–79.79 g·100 g^−1^), protein (7.03–10.03 g·100 g^−1^ dm), fat (4.45–21.30 g·100 g^−1^), ash (0.67–1.33 g·100 g^−1^), and dietary fiber contents (0.03–2.53 g·100 g^−1^) [38,39,40,41].

### 2.3. Total Polyphenols and Antioxidant Activity

The results of antioxidant activity determinations and the amounts of particular polyphenols are presented in Table 4. The content of total polyphenols was in the range of 91.26–256.44 mg·100 g^−1^ dm. The content of total polyphenols was highest in the cookies with the addition of wild roses (256.44 mg) and chokeberries (165.31 mg), while in the cookies with the addition of the other wild-grown fruits (rowans, hawthorns, and elderberries), it was in the range of 139–149 mg (Table 4). The lowest content of these compounds was found in cookies with the addition of sea buckthorn berries (98 mg). It is worth mentioning that all the obtained values were significantly higher than the values obtained in the control cookies (*p* < 0.05). Among the different types of polyphenols, the most abundant in phenolic acids, flavonoids, and anthocyanins were chokeberry cookies together with hawthorn cookies. There were also differences not only in the amount but also in the kind of polyphenolic compounds (Table 4); namely, anthocyanins were detected in only three kinds of cookies: chokeberry, hawthorn, and elderberry (idaein in chokeberry cookies and kuromanine in hawthorn and elderberry cookies). According to the data available in the literature, the total polyphenol content (calculated in mg of gallic acid 100 g^−1^ dm) of cereal-based products and wheat flour was stated in the range of 28.3–344 [42,43]. The results obtained in this study are consistent with this range. In the available literature, there is lack of information on the amount of total polyphenols in wheat-flour cookies with addition of the tested wild-grown fruits. Concerning fruits alone, chokeberry (376–7848 mg) and wild rose fruits (2140–5450 mg) are some of the richest sources of polyphenols (per 100 g), which include anthocyanins, flavonols, flavanols, proanthocyanidins, and phenolic acids [30,31], followed by sea buckthorns (~600 mg/100 g), elderberries (364–582 mg/100 g), rowans (~361.98 mg/100 g), and hawthorns (~285 mg/100 g) [9,10,11,16]. The presented data in the available literature in raw fruits are higher than the content of polyphenols in the cookies baked with addition of different kinds of wild-grown fruits. The baking process at high temperatures (> 200 °C) might have a significant impact on polyphenolic molecules, resulting in some changes in their content and structure [4]. For example, as a result of heat treatment, anthocyanins might be converted to chalcone glycosides and then hydrolyzed to chalcones, which are successively cleaved to form various end products such as aldehydes and acids [44,45]. In addition, metal ions present in food (iron, copper, and manganese) can be involved in the conversion of anthocyanins to quinones, which in turn can react with proteins and other polyphenols to form condensation products which contribute to the browning of food [46]. Flavonoids appear to be more stable during baking. Phenolic acids (protocatechuic, p-hydroxybenzoic, vanillin, salicylic, ellagic, and gallic acids) together with phenylpropanoic acids (p-coumaric, caffeic, ferulic, sinapic) are the main polyphenols in cereals. In cereal kernels, the major phenolic acid is trans-ferulic acid [38]. In wheat grain, insoluble fractions predominantly occur (77%), followed by bound soluble acids (22%), including free and soluble fractions (0.5–1%) [47]. Baking increased the level of free phenolic acids in bread, cakes, and muffins but decreased the amount of bound phenolic acids [48], suggesting that baking may promote the release of phenolic acids, from bound to free. During thermal processing, ferulic acid can be converted to guaiacol, 4-methylguaiacol, 4-ethyl guaiacol, and 4-vinyl guaiacol [49]. This might explain the lack of ferulic acid in the control wheat-flour cookies in this study (Table 3).

The antioxidant activity of the tested cookies was 1.1–15.22 μmol trolox·g^−1^ dm and 2.46–26.12 μmol Fe (II)·g^−1^ dm, as measured by the ABTS and FRAP methods, respectively (Table 3). Independently, using the measuring method, the antioxidant activity of all the fruit-enriched cookies was greater than that of the control cookies (*p* < 0.05). Activity determined by the ABTS method allowed us to rank the examined cookies according to the decreasing value of the discussed parameter as follows: chokeberry > elderberry > rowan and hawthorn > wild rose > sea buckthorn > control. In turn, using the FRAP method, these values were as follows: wild rose > chokeberry > rowan > hawthorn > elderberry > sea buckthorn > control.

The defensive effects of natural antioxidants in fruits and vegetables involve three main groups: phenols, vitamins, and carotenoids. Carotenoids are classified as lipophilic antioxidants, while ascorbic acid and phenols are hydrophilic molecules [50]. The antioxidant activity of berries and berry products can range from 19.0 to 63.1 μmol trolox·g^−1^ dm [51]; however, there are also some superfruits (e.g., wild dried roses or Indian gooseberries) with enhanced antioxidant activity, from 208 to 2615.0 μmol trolox·g^−1^ dm [51]. According to the data available in the literature, the wild-grown fruits had the following antioxidant activity as measured by the ABTS method (μmol trolox·g^−1^): 4.3949 (chokeberries), 38.75 (wild roses), 15.88–63.7 (elderberries), 5.94 (rowanberries), 6.45–9.85 (sea buckthorns), 72.70 (hawthorns); and as measured by the FRAP method (μmol Fe(II)·g^−1^): 185–206.2 (chokeberries), 18.41–81.50; 127.78 (wild roses), 63.7–295.60 (elderberries), 107.5 (rowanberries), 24.8–189.2 (sea buckthorns), and 63 (hawthorns) [7,8,10,13,14,15,52,53]. The observed differences in the antioxidant activity of the examined cookies resulted not only from the analytical method used (ABTS/FRAP) and the differences between fruits, but were also influenced by the antioxidant activity of the individual phenols [54,55]. For example, catechin, epicatechin, rutin, and caffeic acid were characterized by different antioxidant activity, measured by the ability to quench the free radical ABTS, at the level of 3.965, 2.800, 2.074, and 0.965 mol TE/mol respectively, and 0.793, 0.917, 1.156, and 1.018 mol TE/mol, respectively, using the FRAP method [56]. The structure of polyphenolic compounds also plays a significant role [49], namely, the higher the number of hydroxyl groups, the better their antioxidant properties [54]. The para position increases the antioxidant activity compared to the ortho position and meta positions. For instance, caffeic acid (3,4-dihydroxycinnamic acid) with two hydroxyl groups shows better antioxidant properties than p-coumaric acid (4-hydroxycinnamic acid), which has only one hydroxyl group bound to the aromatic ring [48,50,54].

### 2.4. Acrylamide

In the present study, the acrylamide content varied between 81.98 and 1290.77 µg/kg dm, being the highest in the control cookies and lower at a statistically significant level in all the fruit-enriched cookies (*p* < 0.05) (Table 4). For nearly 20 years, the FAO/WHO has been looking for possible ways to reduce high levels of acrylamide in commonly consumed foods such as potatoes and cereal products [1,57,58]. Recently, the Swedish National Food Administration (SNFA) and Stockholm University researchers have reported moderate levels (5–50 µg·1000 g^−1^ dm) and high levels (150–4000 µg·1000 g^−1^ dm) of acrylamide in heat-treated protein-rich foods and carbohydrate-rich foods, respectively [1]. Foods deficient in acrylamide are those which are cooked or nonthermally treated [59]. The occurrence of acrylamide in thermally handled foods represents a worldwide public health concern as a compound which has been categorized as a possible human carcinogen by the International Agency for Research on Cancer (IARC) [1]. The average consumer consumption of acrylamide can vary between countries and dietary habits. It is estimated to be between 0.4 and 1.0 µg/kg bodyweight/day. Other researchers have estimated the acceptable daily intake at 1 µg/kg bodyweight/day, which is an amount exceeded in many common foods [1]. The WHO showed that exposure to low doses may be followed by a period of silent symptoms in which the harmful effects of the chemical may not be clinically apparent but morphological and/or biochemical changes may be present [1,2,3,60,61,62]. The tolerable daily intake of acrylamide for neurotoxicity was estimated at 40 mg/kg/day, while that for cancer—at 2.6 mg/kg/day [1,62]. The Joint Expert Committee on Food Additives reported that the major foods contributing to the total acrylamide intake for most countries are potato crisps (6–46%), potato chips (16–30%), coffee (13–39%), pastry and sweet biscuits (10–20%), and bread (10–30%) [1]. The results of this study clearly indicated that the application of wild-grown fruits lowered the acrylamide content in wheat-flower cookies by 59% (hawthorns), 71% (rowans), 87% (wild roses), 89% (sea buckthorns), 91% (elderberries), and 94% (chokeberries) (Table 3). This phenomenon is possibly caused by the bioactive molecules present in the tested wild fruits. Application of wild-grown fruits creates an opportunity to improve the antioxidant properties of snacks and could contribute to lowering the chemical hazards that naturally develop during the processes of baking at high temperatures (> 120 °C) in the presence of reducing sugars, free amino acids, mainly asparagine, and low moisture at the surface of the food product [1]. The impact of cookie formulation on the acrylamide content has been the subject of different studies [2]. Recently, several strategies have been proposed in order to reduce the high content of acrylamide in cookies: adding calcium salts [57,63], using sucrose instead of reducing sugars such as fructose and glucose [58,64,65], reducing the amount of free asparagine in heated foods by using asparaginase [66,67], diluting the concentration of asparagine by adding glycine [68], and replacing ammonium-containing agents with baking powder [69]. The use of plant antioxidants is also significant. There are both positive and negative effects on acrylamide development [2,70,71]. For example, when the extract, oil, and dried leaves of rosemary were incorporated into wheat dough, acrylamide was reduced by 62%, 67%, and 57%, respectively, compared to the wheat rolls with no rosemary addition [70]. In contrast, grape seed extract added to baked goods had no effect on acrylamide development [71]. Similarly, a different study found that the inclusion of sesamol, vitamin E, and antioxidants such as 2,6-bis (1,1-dimethylethyl)-4-methylphenol into meat before heating led to greater acrylamide production [1]. In another study, chrysin, a flavonoid compound found in different plants, reduced acrylamide-induced neurotoxicity in Wistar rats as a result of its high antioxidant potential [72]. Berry juices (blueberry, black mulberry, and raspberry) significantly recovered the growth of acrylamide-exposed *Saccharomyces cerevisiae* and decreased the level of reactive oxygen species [73]. Addition of wild-grown fruits could be an innovative approach in the production of functional cookies with enhanced antioxidant properties characterized by a reduced level of so-called food-borne toxins (i.e., acrylamide) while maintaining the organoleptic properties acceptable and desirable to potential consumers.

## 3. Materials and Methods

### 3.1. Materials

#### Wheat Cookie Preparation

Fresh samples of mature edible parts of wild-grown fruits (sea buckthorns, elderberries, rowans, chokeberries, wild roses, and hawthorns) were gathered from nonpolluted countryside areas of the Malopolska region (Poland), frozen, and freeze-dried with a lyophilizer (ALPHA 1-4, Martin Christ, Germany). Then, the lyophilizate was minced in a laboratory mincer to obtain a sample not bigger than 0.5 mm (Knifetec Sample Mill 1095, FOSS Tescator, Sweden) and used in that form as a bakery ingredient.

The dough components were as follows (Table 1): wheat flour type 450 (500 g) (”Złote Pola”, Polish Plants Grains S.A., Cracow, Poland), margarine (250 g) (”Kasia”, Unilever, Warsaw, Poland), saccharose (200 g) (”Królewski”, Südzuker Polska S.A., Wroclaw, Poland), eggs (150 g) (“Ale jaja”, poultry firm Woźniak Sp. z o.o., Rawicz, Poland), baking powder (6 g) (Dr. Oetker Poland Sp. z o.o., Gdansk, Poland). Then, 5 g of the corresponding freeze-dried fruits were added to biscuit dough instead of 5 g of wheat flour to each kind of cookie. In the case of the control sample, 5 g of wheat flour were added instead of freeze-dried fruits. After mixing, the ingredients were kneaded into the dough and left in a refrigerator (4 °C, 30 min). After this, the dough was rolled into 7 mm thickness and cut into same-size (3.5 cm) round cookies. These cookies were then baked (200 °C, 8 min) and cooled down. The cookies were stored in tightly closed containers in a dark, dry place. Before each analysis, the biscuits were crushed with a laboratory mincer (Knifetec Sample Mill 1095, FOSS Tescator, Sweden).

### 3.2. Methods

#### 3.2.1. Organoleptic Assessment

Following the Polish and EU standards [74,75,76,77], a panel of 15 trained people assessed the baked cookies. A five-point scale was used, in which shape (0.2), color (0.1), surface (0.15), consistency (0.1), aroma (0.2), and taste (0.25) were taken as quality factors; 5 stands for very good quality; 4—good quality; 3—satisfactory quality; 2—insufficient quality; 1—bad quality; the numbers in parentheses represent the weighting coefficients. Water was available for the panelists in order to flush the mouth between analyses.

#### 3.2.2. Chemical Composition

The dry matter (dm) content of the wheat cookies, as well as the amounts of protein, fat, and ash were measured using the standard methods recommended by the Association of Official Analytical Chemists (AOAC) [78]. Moisture percentage was determined by the oven dry method (AOAC method 940.26) using a laboratory drier (SML 30/250, Zalmed, Warszawa, Poland); ash was determined by incineration (550 °C) of known weights of the samples in a muffle furnace (FCF5SH, Czylok, Jastrzębie-Zdrój, Poland) (AOAC method 930.05); crude fat was determined by the Soxtec automated extraction method (AOAC method 930.09) [78] using a Soxtec Avanti’s 2050 Auto Extraction Foss Unit (Tecator, Hillerød, Sweden); protein (N × 6.25) was determined by the Kjeldahl method using a FOSS Digester and Autodistillation Unit, KjeltecTM 8200 (Tecator Foss, Hillerød, Sweden) (AOAC method 978.04); the total dietary fiber was determined with a commercial kit (K-TDFR, Megazyme International Ireland, Bray Business Park, Bray, Co. Wicklow, Ireland). The carbohydrate content was determined by difference: 100 − (moisture + ash + protein + fat).

#### 3.2.3. Preparation of Methanol–Acetone Extracts

Fresh samples (5 g) were extracted with a mixture of 80% acidified methanol (methanol–HCl, 2% (95%/5% *v*/*v*) (Sigma-Aldrich, St. Louis, MO, USA)) for 2 h at 25 °C, then centrifuged (1500 g, 15 min). The supernatant was saved and the sediment was extracted again with 40 mL solution of 70% acetone (POCH, Gliwice, Poland) for the next 2 h and then centrifuged (1500 g, 15 min). The supernatants from both centrifugations were combined and used for further analysis.

#### 3.2.4. Total Polyphenols

The total polyphenols in the tested cookies were determined by the method described by Swain and Hillis [79] with the use of the Folin–Ciocalteu reagent (Sigma-Aldrich, St. Louis, MO, USA). Previously prepared methanol–acetone extracts (3.2.3.) were incubated with the Folin–Ciocalteu reagent at room temperature (25 °C), and the absorbance was read at 760 nm in a spectrophotometer (Spectro 2000RS, Labomed, Inc. Los Angeles, CA, USA). The concentration of polyphenols was expressed in mg of gallic acid per 100 g dm.

#### 3.2.5. Determination of ABTS Activity

The antioxidant activity of the tested cookies was determined using the method described by Re et al. [80] with the use of ABTS (2,2-azinobis) (3-ethyl-beznothaizoline-6-sulfonic acid) (Sigma-Aldrich, St. Louis, MO, USA). The 0.5 mL aliquot of methanol–acetone extracts (3.2.3.) were made up to 1 mL with methanol; then 2 mL of the free radical ABTS solution was added. The mixture was incubated at 30 °C for 6 min. The absorbance of the solution was measured in a spectrophotometer (Spectro 2000RS, Labomed, Inc., Los Angeles, CA, USA) at 734 nm.

#### 3.2.6. Determination of FRAP Activity

The FRAP (ferric-reducing ability of plasma) assay was performed according to the adapted method of Benzie and Strain [81] with respect to its latest modification by Bartoń, Fołta, and Zachwieja [82]. The working reagent of FRAP was prepared as a mixture of (1) acetic buffer (pH = 3.6), (2) TPTZ (2,4,6-Tris(2-pyridyl)-s-triazine) (Sigma-Aldrich, St. Louis, MO, USA), and (3) ferric trichloride hexahydrate (FeCl_3_·6H_2_O) (Sigma-Aldrich, St. Louis, MO, USA) in the following ratio: 2:1:1. Ferric (FeIII)-to-ferrous (FeII) ion reduction created an intensive blue color of the FeII–TPTZ complex with the absorbance maximum at 593 nm. A Multi-Detection Microplate Reader (Synergy 2, BioTek Instruments, Inc., Winoosoki, VT, USA) was used to perform analysis. The assay was conducted at 37 °C. An acetate buffer of 0.400 mL was dispensed into each well while 0.050 mL of the methanol–acetone extracts (3.2.3.) were pipetted, and 0.2 mL of the working FRAP reagent were then added. Absorbance was measured at ca. 1.5 min intervals within 60 min after addition of the working FRAP reagent. The method was calibrated by the use of the ferrous sulfate heptahydrate (Fe_2_SO_4_·7H_2_O) (Sigma-Aldrich, St. Louis, MO, USA) standard solution at the concentration range of 0–1.5 mmol·L^−1^.

#### 3.2.7. Reversed-Phase High-Performance Liquid Chromatography (RP-HPLC) Analysis of Flavonoids and Phenolic Acids

Sixty grams (60 g) of dry mass were extracted with 10 mL of methanol in an ultrasonic bath (POLSONIC Palczyński Sp. J., Warsaw, Poland) for 1 h at 30 °C. Phenolic acids and flavonoids in the dry biomass were quantified in methanol extracts (sonication, 30 °C, 1 h). RP-HPLC analyses were conducted according to Ellnain-Wojtaszek and Zgórka [83] with our modification on a Merck–Hitachi liquid chromatograph (LaChrom Elite, Hitachi, Tokyo, Japan) equipped with diode array detector (DAD) L-2455 and a Purospher^®^ RP-18e (250 × 4 mm/5 mm) column. The analyses were carried out at 25 °C, with a mobile phase consisting of methanol (A) and methanol: 0.5% acetic acid at a 1:4 ratio (*v*/*v*) (B). The gradient was as follows: 100% B for 0–20 min; 100–80% B for 20–35 min; 80–60% B for 35–55 min; 60–0% B for 55–70 min; 0% B for 70–75 min; 0–100% B for 75–80 min; 100% B for 80–90 min at a flow rate of 1 mL/min^−1^, λ = 254 nm (phenolic acids, catechins), λ = 370 nm (flavonoids). Identification was performed by comparison of the retention times of the peaks with authentic reference compounds and co-chromatography with standards. Quantification was performed by measurement of the peak area with reference to the standard curve derived from five concentrations (0.03125–0.5 mg/mL^−1^). The standards were as follows: caffeic, chlorogenic, cinnamic, gallic, gentisic, o-coumaric, protocatechuic, salicylic, sinapic, syringic acids, apigetrin (apigenin 7-glucoside), hyperoside (quercetin 3-galactoside), isorhamnetin, kaempferol, luteolin, populnin (kaempferol 7-O-glucoside), quercetin, quercitrin, rhamnetin, rutin, vitexin (Sigma-Aldrich, St. Louis, MO, USA); p-coumaric, vanillic, ferulic, p-hydroxybenzoic acids (Fisher Scientific, Bishop Meadow Road, Loughborough, Leicestershire, LE11 5RG), catechin, epigallocatechin, epicatechin gallate, epicatechin, epigallocatechin gallate, cynaroside (luteolin 7-O-glucoside) (ChromaDex,10005 Muirlands Blvd., Suite G, Irvine, CA, 92618, USA).

#### 3.2.8. High-Performance Liquid Chromatography (HPLC) Analysis of Anthocyanins

Sixty grams (60 g) of dry mass were extracted with 10 mL of methanol containing 5% (*v*/*v*) formic acid in an ultrasonic bath (POLSONIC Palczyński Sp. J., Warsaw, Poland) for 1 h at 30 °C. After procurement, the extracts were strained through a 0.2 µm filter and analyzed using the HPLC-DAD method. RP-HPLC analyses were conducted according to Weber et al. [84] with our modification on a Merck–Hitachi liquid chromatograph (LaChrom Elite, Hitachi, Tokyo, Japan) equipped with DAD L-2455 and a Purospher^®^ RP-18e (250 × 4 mm/5 mm) column. Analyses were carried out at 25 °C, with a mobile phase consisting of acetonitrile (A) and 0.1% formic acid (B). The gradient was as follows: 95–80% B for 0–15 min; 80–70% B for 15–20 min; 70% B for 20–25 min; 70–10% B for 25–30 min; 10% B for 30–35 min; 10–95% B for 35–45 min; 95% B for 45–55 min at a flow rate of 1 mL/min^−1^, λ = 520 nm. Identification was performed by comparison of the retention times of the peaks with authentic reference compounds and co-chromatography with standards. Quantification was performed by measurement of the peak area with reference to the standard curve derived from five concentrations (0.03125–0.5 mg/mL^−1^). The standards were as follows: callistephin chloride, cyanidin chloride, idaein chloride, keracyanin chloride, kuromanin chloride, malvidin chloride, malvidin-3-O-galactoside chloride, malvin chloride, oenin chloride, pelargonidin chloride, pelargonin chloride (Sigma-Aldrich, St. Louis, MO, USA).

#### 3.2.9. Determination of the Acrylamide Content

The acrylamide content in the prepared samples was determined by HPLC. The analyses were performed using a Knauer chromatograph with a UV–Vis detector (KNAUER Wissenschaftliche Geräte GmbH, Berlin, Germany) and the chromatographic separation parameters were as follows: eluent: water/acetonitrile (8:2 *v*/*v*); flow rate: 0.6 mL/min; column: Eurokat H + ion exchange, 20 μL loop; wavelength: 200 nm. The samples for chromatographic analyses were prepared based on the method of Paleologos and Kontaminas [85] using aqueous extraction and deprotection employing the Carrez I and Carrez II solutions (Sigma-Aldrich, St. Louis, MO, USA) and degreasing with hexane. The cookie samples were weighed and the prepared solutions were strained through a syringe filter and subjected to chromatographic analyses.

### 3.3. Data Analyses

The results were presented as ranges of at least three parallel repetitions with a standard deviation around the mean. One-way analysis of variance was applied in order to assess the influence of different wild-grown fruit additions on the tested parameters. The Duncan test was used in order to evaluate the significance of differences at a level of *p* < 0.05. All the calculations were carried out using Statistica v.10 software (Statsoft, Inc., Tulsa, OK, USA).

## 4. Conclusions

The overall ratings of the tested cookies with the addition of chokeberries, hawthorns, sea buckthorns, and elderberries had a more than satisfactory grading, while wild rose and rowan cookies were the most widely accepted and best rated by the panelists. All the fruit-enriched cookies had significantly higher antioxidative properties (*p* < 0.05) in comparison to the control cookies. There were differences in quality and quantity of particular polyphenols among them. The acrylamide content of all the fruit-enriched cookies was significantly decreased, although the particular variants differed between each other (*p* < 0.05). In this regard, cookies enriched with wild-grown fruits could constitute a promising novel snack food. Some effort should be taken in order to improve the properties of hawthorn, chokeberry, sea buckthorn, and elderberry cookies, especially the organoleptic properties. This is an issue that requires thorough investigation, and the obtained results confirm the need to continue research. It could also be beneficial to investigate other functional properties of the designed cookies, especially their influence on the glycemic response, which could be advantageous for people suffering from type 2 diabetes and those who would like to protect themselves against development of this disease. The continuation of the research is relevant regarding the influence of the designed cookies on the glycemic index and the bioavailability of the polyphenolic compounds tested on human volunteers.

## Figures and Tables

**Table 1:** The recipe formulation of different kinds of cookies.

	Dough Ingredients (g)					
Cookies Kinds	Wheat Flour	Margarine	Saccharose	Eggs	Baking Powder	Lypholized Fruits
**Control**	500	250	200	150	5	0
**Chokeberry** (*Aronia melanocarpa*)	495	250	200	150	5	5
**Wild rose** (*Rosa canina* L.)	495	250	200	150	5	5
**Elderberry** (*Sambucus nigra* L.)	495	250	200	150	5	5
**Hawthorn** (*Crataegus* L.)	495	250	200	150	5	5
**Rowan** (*Sorbus aucuparia* L.)	495	250	200	150	5	5
**Sea buckthorn** (*Hippophae rhamnoides* L.)	495	250	200	150	5	5

**Table 2:** Organoleptic characteristics of the cookies enriched with wild-grown fruits.

	Cookies with Fruits						
	Chokeberry (*Aronia melanocarpa*)	Wild rose (*Rosa canina* L.)	Elderberry (*Sambucus nigra* L.)	Hawthorn (*Crataegus* L.)	Rowan (*Sorbus aucuparia* L.)	Sea buckthorn (*Hippophae rhamnoides* L.)	Control
Quality parameters							
Appearance	0.80 ^a^ ± 0.1	0.93 ^b^ ± 0.1	0.80 ^a^ ± 0.1	0.72 ^a^ ± 0.1	0.93 ^b^ ± 0.1	0.80 ^a^ ± 0.1	0.91 ^b^ ± 0.1
Surface	0.60 ^bc^ ± 0.1	0.67 ^d^ ± 0.1	0.55 ^a^ ± 0.1	0.55 ^a^ ± 0.6	0.60 ^abc^ ± 0.1	0.57 ^ab^ ± 0.1	0.65 ^cd^ ± 0.1
Consistency	0.40 ^abc^ ± 0.1	0.42 ^c^ ± 0.1	0.37 ^ab^ ± 0.0	0.35 ^a^ ± 0.6	0.40 ^bc^ ± 0.1	0.37 ^ab^ ± 0.1	0.39 ^abc^ ± 0.0
Color	0.41 ^a^ ± 0.0	0.46 ^b^ ± 0.1	0.40 ^a^ ± 0.1	0.40 ^b^ ± 0.0	0.45 ^b^ ± 0.1	0.40 ^a^ ± 0.0	0.43 ^ab^ ± 0.0
Flavor	0.79 ^ab^ ± 0.1	0.85 ^b^ ± 0.1	0.75 ^ac^ ± 0.1	0.68 ^c^ ± 0.1	0.83 ^ab^ ± 0.1	0.76 ^a^ ± 0.1	0.80 ^ab^ ± 0.1
Tastiness	0.90 ^a^ ± 0.1	1.01 ^b^ ± 0.4	0.85 ^c^ ± 0.2	0.75 ^c^ ± 0.2	0.97 ^ab^ ± 0.2	0.88 ^a^ ± 0.1	0.97 ^ab^ ± 0.2
**Total**	3.89 ^c^ ± 0.3	4.40 ^a^ ± 0.4	3.71 ^c^ ± 0.4	3.45 ^d^ ± 1.3	4.19 ^b^ ± 0.4	3.79 ^c^ ± 0.3	4.14 ^b^ ± 0.4

The results were presented as the means ± SD (standard deviation). The values with different letters in rows are significantly different at *p* < 0.05.

**Table 3:** Chemical composition of the tested cookies.

Analysed Parameters/Cookies with Fruits	Chokeberry (*Aronia melanocarpa*)	Wild Rose (*Rosa canina* L.)	Elderberry (*Sambucus nigra* L.)	Hawthorn (*Crataegus* L.)	Rowan (*Sorbus aucuparia* L.)	Sea Buckthorn (*Hippophae rhamnoides* L.)	Control
Dry matter (%)	95.50 ± 0.1 ^c^	94.39 ± 0.1 ^a^	94.91 ± 0.1 ^b^	94.83 ± 0.2 ^c^	94.73 ± 0.1 ^c^	94.75 ± 0.1 ^c^	94.18 ± 0.2 ^a^
Carbohydrates (g·100 g^−1^ dm)	65.31 ± 0.0 ^a^	60.62 ± 0.1 ^f^	62.55 ± 0.0 ^b^	59.26 ± 0.1 ^c^	61.34 ± 0.0 ^d^	56.86 ± 0.2 ^e^	60.07 ± 0.0 ^f^
Proteins (g·100 g^−1^ dm)	9.09 ± 0.0 ^d^	9.71 ± 0.0 ^c^	9.57 ± 0.0 ^b^	9.27 ± 0.0 ^a^	9.30 ± 0.0 ^a^	9.53 ± 0.0 ^b^	9.76 ± 0.1 ^c^
Fat (g·100 g^−1^ dm)	20.44 ± 0.1 ^ab^	21.03 ± 0.1 ^c^	20.39 ± 0.1 ^a^	20.56 ± 0.1 ^b^	21.00 ± 0.1 ^c^	21.22 ± 0.1 ^d^	21.17 ± 0.1 ^d^
Dietary fiber (g·100 g^−1^ dm)	3.12 ± 0.0 ^d^	2.19 ± 0.1 ^a^	1.69 ± 0.1 ^c^	5.00 ± 0.1 ^e^	2.27 ± 0.1 ^ab^	6.36 ± 0.1 ^f^	2.33 ± 0.0 ^b^
Ash (g·100 g^−1^ dm)	0.66 ± 0.1 ^c^	0.84 ± 0.0 ^ab^	0.71 ± 0.0 ^d^	0.74 ± 0.0 ^e^	0.82 ± 0.0 ^a^	0.78 ± 0.0 ^f^	0.85 ± 0.0 ^b^

The results were presented as the means ± SD. The values with different letters in rows are significantly different at *p* < 0.05.

**Table 4:** Total polyphenols, polyphenolic compounds’ profile, antioxidant activity, and acrylamide content of the tested cookies.

	Chokeberry (*Aronia melanocarpa*)	Wild Rose (*Rosa canina* L.)	Elderberry (*Sambucus nigra* L.)	Hawthorn (*Crataegus* L.)	Rowan (*Sorbus aucuparia* L.)	Sea Buckthorn (*Hippophae rhamnoides* L.)	Control
**Total polyphenols** **(mg·100 g^−1^ dm)**	165.31 ± 1.2 ^b^	256.44 ± 1.0 ^a^	144.69 ± 0.5 ^c^	139.52 ± 0.7 ^e^	149.66 ± 1.6 ^d^	98.66 ± 0.4 ^f^	91.26 ± 1.50 ^g^
**Phenolic acids (µg·100 g^−1^ dm)**							
Neochlorogenic acid	919.28 ± 4.85 ^a^	nd	nd	178.03 ± 0.14 ^b^	5366.52 ± 12.11 ^c^	nd	nd
Protocatechuic acid	269.20 ± 0.62 ^a^	59.33 ± 0.29 ^b^	131.23 ± 0.72 ^c^	33.59 ± 0.29 ^d^	2244.69 ± 0.90 ^c^	nd	23.72 ± 0.01 ^e^
Chlorogenic acid	1026.18 ± 4.94 ^a^	nd	nd	113.59 ± 1.23 ^b^	nd	51.00 ± 0.87 ^d^	nd
Cinnamic acid	3.32 ± 1.85 ^a^	nd	nd	nd	77.18 ± 0.04 ^b^	nd	nd
Caffeic acid	nd	nd	nd	nd	nd	nd	nd
Vanillic acid	nd	nd	nd	nd	nd	nd	42.71 ± 0.18
**Flavanols (µg·100 g^−1^ dm)**							
Catechin	21.82 ± 1.23 ^ab^	45.60 ± 20.17 ^b^	14.05 ± 0.12 ^a^	23.73 ± 1.23 ^ab^	nd	11.70 ± 0.52 ^a^	nd
Epicatechin	792.48 ± 0.53 ^a^	nd	nd	823.86 ± 6.58 ^b^	nd	nd	nd
**Flavonols (µg·100 g^−1^ dm)**							
Quercetin	202.66 ± 1.71 ^a^	163.82 ± 0.29 ^b^	156.04 ± 0.23 ^c^	133.01 ± 0.07 ^d^	19.05 ± 0.18 ^e^	200.73 ± 0.76 ^f^	nd
Quercetin-3-galactoside (hyperoside)	237.19 ± 3.17 ^a^	156.33 ± 0.98 ^b^	nd	309.04 ± 0.47 ^c^	77.18 ± 0.45 ^d^	190.86 ± 1.79 ^e^	nd
Kaempferol-7-O-glucoside (populnin)	nd	743.44 ± 17.35	nd	nd	nd	nd	nd
Rutin	461.74 ± 10.92 ^a^	100.60 ± 5.05 ^b^	1083.20 ± 1.40 ^c^	133.04 ± 0.08 ^d^	126.34 ± 0.80 ^e^	304.96 ± 2.06 ^f^	nd
**Flavones (µg·100 g^−1^ dm)**							
Apigenin-8-C-glucoside (vitexin)	nd	nd	nd	166.59 ± 2.19	nd	nd	nd
Luteolin 7-O-glucoside (cynaroside)	nd	nd	nd	nd	nd	294.36 ± 0.26	nd
**Anthocyanins (µg·100 g^−1^ dm)**							
Cyanidin-3-glucoside (idaein)	78.05 ± 0.36	nd	nd	nd	nd	nd	nd
Cyanidin-3-O-glucoside (kuromanine)	nd	nd	376.1 ± 11.42 ^a^	279.95 ± 3.31 ^b^	nd	nd	nd
**Antioxidant activity**							
ABTS (µmol·g^−1^ dm)	15.22 ± 0.05 ^a^	5.38 ± 0.04 ^d^	9.42 ± 0.01 ^b^	7.58 ± 0.01 ^c^	7.61 ± 0.05 ^c^	4.99 ± 0.03 ^e^	1.11 ± 0.00 ^f^
FRAP (µmol·g^−1^ dm)	17.47 ± 0.05 ^b^	26.12 ± 0.83 ^a^	11.37 ± 0.17 ^e^	12.30 ± 0.17 ^d^	13.66 ± 0.16 ^c^	9.83 ± 0.27 ^f^	2.46 ± 0.08 ^g^
**Acrylamide content** **(µg·1000 g^−1^ dm)**	81.98 ± 0.95 ^a^	173.90 ± 0.54 ^d^	120.26 ± 1.09 ^b^	524.96 ± 1.98 ^f^	370.63 ± 1.76 ^e^	136.06 ± 0.65 ^c^	1290.77 ± 1.23 ^g^

The results were presented as the means ± SD. The values with different superscript letters in the same row are significantly different at *p* < 0.05; nd—not detected.

## Data Availability

The data that support the findings of this study are available from the corresponding author upon reasonable request.
